# Experimental Test of Connector Rotation during DNA Packaging into Bacteriophage ϕ29 Capsids

**DOI:** 10.1371/journal.pbio.0050059

**Published:** 2007-02-20

**Authors:** Thorsten Hugel, Jens Michaelis, Craig L Hetherington, Paul J Jardine, Shelley Grimes, Jessica M Walter, Wayne Falk, Dwight L Anderson, Carlos Bustamante

**Affiliations:** 1 Department of Physics, Technical University, Munich, Germany; 2 Munich Center for Integrated Protein Science and Center for NanoScience, Munich, Germany; 3 Department of Chemistry and Biochemistry, Ludwig-Maximilians-University, Munich, Germany; 4 Department of Physics, University of California, Berkeley, California, United States of America; 5 Lawrence Berkeley National Laboratory, Berkeley, California, United States of America; 6 Department of Diagnostic and Biological Sciences, University of Minnesota, Minneapolis, Minnesota, United States of America; 7 Department of Microbiology, University of Minnesota, Minneapolis, Minnesota, United States of America; 8 Howard Hughes Medical Institute, University of California, Berkeley, California, United States of America; Adolf-Butenandt-Institut, Germany

## Abstract

The bacteriophage ϕ29 generates large forces to compact its double-stranded DNA genome into a protein capsid by means of a portal motor complex. Several mechanical models for the generation of these high forces by the motor complex predict coupling of DNA translocation to rotation of the head-tail connector dodecamer. Putative connector rotation is investigated here by combining the methods of single-molecule force spectroscopy with polarization-sensitive single-molecule fluorescence. In our experiment, we observe motor function in several packaging complexes in parallel using video microscopy of bead position in a magnetic trap. At the same time, we follow the orientation of single fluorophores attached to the portal motor connector. From our data, we can exclude connector rotation with greater than 99% probability and therefore answer a long-standing mechanistic question.

## Author Summary

The life cycles of many viruses include a self-assembly stage in which a powerful molecular motor packs the DNA genome into the virus's preformed shell (the capsid). Biochemical and biophysical studies have identified essential components of the packaging machinery and measured various characteristics of the packaging process, while crystallography and electron microscopy have provided snapshots of viral structure before and after packaging. In bacteriophage ϕ29 assembly, the DNA passes into the shell through a channel formed by a structure called the connector. Structurally motivated models over the past 30 years have coupled DNA movement to rotation of the connector relative to the capsid. We describe a direct test of the connector rotation hypothesis, combining magnetic single-molecule manipulation techniques and single-molecule fluorescence spectroscopy. In our experiments, we use a single-dye molecule attached specifically to the connector as a reporter for its orientation and simultaneously observe the translocation of a magnetic bead attached to the DNA that is being packaged. From our data, we can exclude connector rotation with greater than 99% probability and therefore answer a long-standing mechanistic question.

## Abstract 3

dsDNA compaction into bacteriophage capsids is observed in packaging complexes. Unlike in previous models, this compaction is found not to be driven by a rotating motor complex.

## Introduction

As part of its viral infection cycle, the Bacillus subtilis bacteriophage ϕ29 packages its double-stranded DNA genome into a preformed capsid shell, or prohead, by means of a powerful molecular motor [1,2]. The DNA-packaging motor is situated at a unique 5-fold vertex of the prohead and is a complex assembly of multiple components. At the core of the motor is the dodecameric head-tail connector, gene product 10 (gp10). Associated with the connector is a ring of RNA molecules (prohead RNA or pRNA), which is required for packaging. A ring of ATPases (gp16) interacts with the pRNA to complete the packaging machinery. gp16 belongs to the Her A, FtsK superfamily of ATPases [3]. Hydrolysis of ATP powers the motor and drives viral DNA into the prohead.

While numerous biochemical, structural, biophysical, and theoretical studies have elucidated many details of the packaging process [1,2,4–15], a complete mechanistic understanding of how the components of the portal motor force the DNA into the capsid has not been presented. In particular, many theoretical models for the function of the connector have been proposed [5,7,16–18]. Most of these models include a rotation, either passive or active, of the connector with respect to the prohead shell—an idea first introduced by Hendrix in 1978 [16]. Recently, a study of DNA packaging in T4, using cross-linking of bulky domains to the connector that could interfere with connector rotation, provided indirect evidence that the connector of T4 does not rotate during packaging [19]. However, no direct structural, biochemical, or biophysical experiments have been published that address the rotation hypothesis. Here, we directly test this hypothesis using single-molecule fluorescence polarization (SMFP) spectroscopy in combination with single-molecule force spectroscopy.

Single-molecule force spectroscopy has proven to be a powerful method for studying the movement of motor proteins. In recent years, a wealth of different systems has been studied, such as actin- and microtubule-based molecular motors [20,21]; motors that move along DNA, like DNA polymerase [22], RNA polymerase [23–26], exonuclease [27,28], and DNA pumps [2,29]; polymerization motors [30]; as well as motors that move pili [31] or whole bacteria [32]. Here, we use a magnetic tweezers set-up to observe the packaging of many DNA-packaging complexes simultaneously.

Single-molecule fluorescence spectroscopy has been used recently to study conformational changes of single-motor complexes [33–39]. In particular, detection of changes in the polarization of emission by a single-dye molecule is well suited to investigate conformational changes involving rotation events [33,34,37]. We utilize this method to investigate the putative rotation of the connector protein with respect to the prohead shell.

To test the hypothesis of connector rotation, we track orthogonal polarization components of fluorescent light emitted by single-dye molecules attached to the connector. Several issues must be overcome to ensure experimental fidelity: (i) DNA packaging by the labeled motor complex must be observed simultaneously with the fluorescence detection; (ii) dye labeling must be specific to the connector; (iii) the dye molecule must stay at a fixed angle relative to the connector; and (iv) the prohead itself must be immobilized without possibility of rotation. In order to fulfill all these requirements, we designed and implemented a combined SMFP and magnetic tweezers packaging assay as described below. The results of our experiment allow us to rule out connector rotation during packaging with more than 99% probability.

## Results

### Single-Molecule Packaging

**Figure 1:**
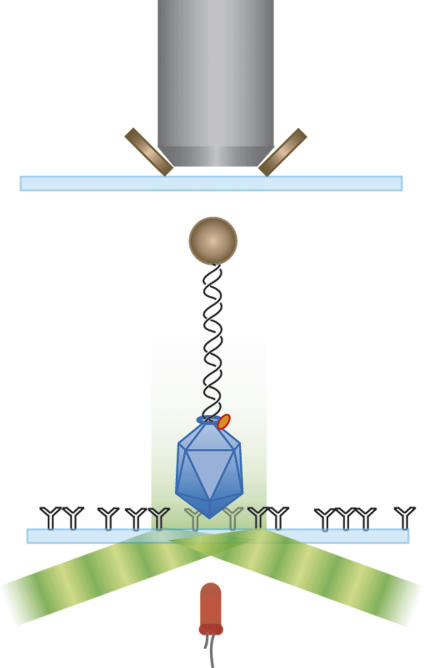
Schematic of the Experimental Geometry (Not to Scale) Stalled-packaging complexes are attached to the surface of the flow-chamber via biotinylated antibodies to the prohead major capsid protein, gp8. The dye molecule, attached to the connector, is excited via an evanescent wave using total internal reflection. The biotinylated free end of the DNA is attached to streptavidin-coated superparamagnetic beads that are pulled away from the fluorescence excitation by a magnetic field gradient that is created by a pair of magnets next to the objective. The magnetic beads are illuminated for video microscopy using a red LED; both the signals for bright-field and fluorescence images are collected by a high NA microscope objective.

Figure 1 shows a schematic of the experimental geometry. Packaging is initiated in bulk as described previously [2], and complexes stalled with non-hydrolyzable ATP analog (ATP-γS) and enriched for active particles are bound to streptavidin-coated superparamagnetic beads via a biotin modification to the distal end of the DNA (see Materials and Methods for details). Antibodies against the capsid protein gp8 are used to anchor the stalled complexes via the prohead shell to a quartz slide. The experiment is observed through a glass cover slip, which together with the quartz slide forms a fluid chamber, using a 1.2-numerical aperture (NA) water-immersion objective. Magnets placed on either side of the objective pull the beads away from the quartz surface, stretching the unpackaged DNA under a force of about 0.1 pN. We monitor the beads via video microscopy and can calculate the length of the DNA tether through either Brownian motion [40] or change of focus of the bead image [41]. Upon exchange of buffers to remove ATP-γS and reintroduce ATP, the complexes resume packaging as confirmed by gradual reduction of tether length. The packaging is monotonic and ATP-dependent. In a typical experiment, about 80% of stalled, tethered complexes package to completion at rates consistent with previous bulk and single-molecule measurements [8].

### Single-Molecule Labeling

Conformational changes that occur during the enzymatic cycle of a motor can be efficiently probed by attaching dye molecules as local reporters to specific proteins of a large multi-subunit complex. Single-dye molecules are sufficiently small so as not to interfere sterically with biological activity in most cases. The simplest way to attach a dye molecule as a reporter to a specific site of a protein is to make use of the high specificity of a cysteine-maleimide reaction. To this end, one needs to make point mutations in order to remove native cysteines, which could be inadvertently labeled, and introduce new cysteines (one at a time) to desired exposed locations. A dye molecule with a reactive maleimide group can then be covalently attached to that site.

**Figure 2:**
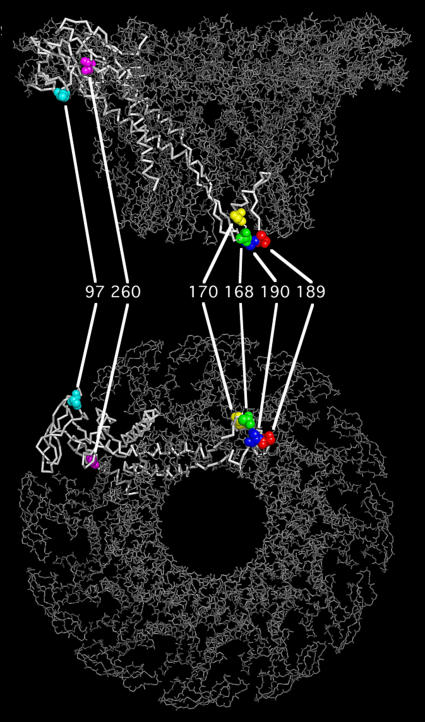
Connector Structure with Position of Dye Labels A schematic of the structure of the connector, based on the crystal structure by Simpson et al. [5] is shown. The positions of the residues that were mutated to cysteines and investigated with single-molecule fluorescence are indicated.

**Figure 3:**
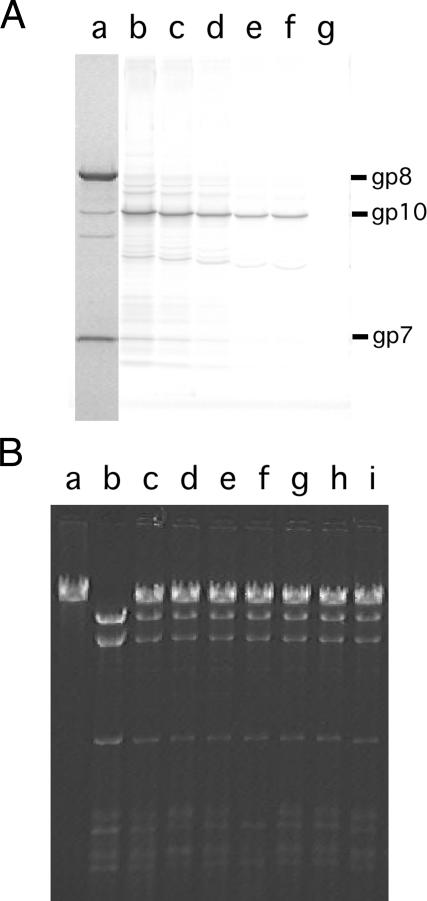
Dye Labeling and DNA Packaging of ϕ29 Proheads (A) SDS-PAGE of 170C-connector-mutant proheads. Protein stain of proheads shows the structural components gp8 (capsid), gp8.5 (fiber), gp10 (connector), and gp7 (scaffold) in lane (a). Fluorescence scan of the gel showing labeled proheads with various amounts of dye per gp10 monomer used in labeling reaction: 1 dye per gp10, lane (b); 0.5 dyes per gp10, lane (c); 0.25 dyes per gp10, lane (d); 0.125 dyes per gp10, lane (e); 0.0625 dyes per gp10, lane (f); and no dye, lane (g). The bands in the fluorescence scan with no match in the protein stain originate from highly reactive but quantitatively minor E. coli proteins. (B) DNA packaging tested by nuclease (EcoRI) protection assay using the labeled proheads from (A). Lane (a) shows input DNA-gp3; lane (b) shows a negative (no ATP) control. Packaged DNA is protected from nuclease digestion. Packaging activity is unaffected by dye labeling when compared to a 193C packaging control, lane (c). (B) Shows labeled proheads from (A), ranging from 1 dye per gp10, lane (d); 0.5 dyes per gp10, lane (e); through to no dye, lane (i).

For our experiments, we replaced the two native cysteines of the connector with serines by site-directed mutagenesis and introduced new cysteines at various positions at the inside and outside of the connector. Figure 2 shows the X-ray crystallographic structure of the connector highlighting the position of the amino acids that were mutated to cysteines (see Materials and Methods). Since current techniques are unable to assemble the free connector protein gp10 into a prohead in vitro, it was necessary to label the connector in the presence of the complete capsid, including capsid (gp8), head fiber (gp8.5), and scaffold proteins (gp7) (none of which contain cysteines). Figure 3A shows, as an example, the fluorescence image of a denaturing gel (SDS-PAGE) of the proheads with a cysteine at amino acid 170 (170C) of the connector, labeled with a Cy3-maleimide. About 80% of the total fluorescence signal is in the single band of the connector, while capsid protein (which makes up more than 95% of the total mass), head fibers, scaffolding protein, and residual contaminants of the Escherichia coli particle expression system show only very weak Cy3 fluorescence. Furthermore, the labeled proheads package DNA in vitro with the same efficiency as unlabeled particles, as can be seen by bulk packaging experiments (see Figure 3B and Materials and Methods).

Having established a specific labeling scheme in a complex that is competent for in vitro packaging, we also need to ensure that the dye molecule can act as a reporter for a rotational movement of the connector. Several criteria must be satisfied: the dye must be bright enough to allow integration times less than one rotational period; the time before photobleaching of the dye molecule must be long enough to observe several rotations; the dye must be attached at angles relative to the connector axis and relative to the objective optical axis such that changes in the dye dipole moment can be seen with our instrument; and the dye molecule must be attached rigidly. To address the latter concern, we measured the fluorescence anisotropy of an ensemble of labeled proheads. The resulting anisotropies of the labeled mutant particles were typically *r* ∼0.3. The common interpretation is that the dye can freely rotate within a cone that is defined by steric limitations; in this case, the measured anisotropy corresponds to a cone half-angle of around 25°. However, the bulk anisotropy measurements only test the freedom of the dye molecules to reorient on the time scale of the fluorescence lifetime (a few nanoseconds). In order to measure the time evolution of the orientation of a connector monomer (a few seconds) and to probe the time before photobleaching of the dye, as well as its brightness and orientation, we had to perform single-molecule fluorescence experiments.

### Single-Molecule Fluorescence

**Figure 4:**
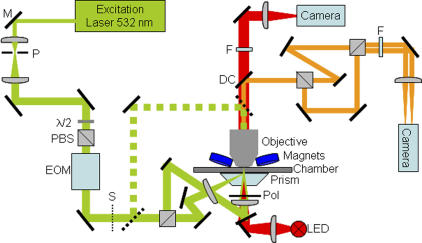
Schematic of the Experimental Setup Combined SMFP and magnetic tweezers setup. M, mirror; P, pinhole; λ/2, λ/2 plate; PBS, polarizing beam splitter; EOM, electro optical modulator; S, shutter; F, filter; Pol, polarizer; DC, dichroic mirror. Dashed components can be removed and are solely used for alignment purpose.

The long-term rotational rigidity of the dye, the rigidity of immobilization of the packaging prohead complexes, and the suitability of the dye orientation relative to the rotation axis can all be studied in single-molecule experiments [42]. To this end, we measured the polarization of the emitted fluorescence from proheads labeled with single-dye molecules. The labeled, stalled prohead complexes are attached to the surface of the micro-fluidic chamber (see Materials and Methods) and illuminated in prism-type total internal reflection geometry, with a green laser (λ = 532 nm). A schematic of the experimental setup is shown in Figure 4 (see also Materials and Methods). The fluorescence signal is collected by the objective and spatially separated into two perpendicular polarization components that are simultaneously imaged on a charge-coupled device camera. An overlay of the two polarization images yields a time-dependent fluorescence signal for each fluorophore in two orthogonal polarizations, thus allowing us to track the changes in relative orientation of a single dye in real time.

**Figure 5:**
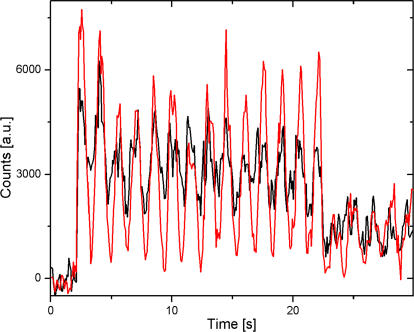
Fluorescence Polarization Studies of Dye-Labeled, Stalled-Packaging Complexes Dye-labeled, stalled-packaging complexes were attached to the surface of a flow chamber and excited using the total internal reflection microscope. The excitation polarization was rotated between s- and p-polarization with a frequency of 0.7 Hz. The emitted fluorescence was separated into s- and p-polarization, respectively, and simultaneously detected (black and red). The dye bleached after 22 s. The integration time per data point was 75 ms. a.u., arbitrary units.

ϕ29 packaging complexes stalled by incubation with ATP-γS and therefore incapable of enzymatically driven rotation were used to assess the dye suitability. Figure 5 shows the fluorescence signal of a single fluorophore attached to a stalled complex, with the excitation polarization being rotated between the two orthogonal directions, henceforth called horizontal and vertical, with a frequency of 0.7 Hz (see Figure 5 for details). Fluorescence signals were integrated for 75 ms, and a typical time before photobleaching of the dye molecule was 10 s. The measured intensities in the two emission channels (perpendicular polarizations) are shown in black and red. The change in excitation polarization can be seen through an oscillation of the intensity in each channel. We observed a correlated signal (which is a very clear indication for a stable orientation of the dye) in more than 50% of the test experiments. Furthermore, it is important to note that the average signal intensity in the two channels remains almost constant over the lifetime of the dye, which shows that the dye keeps a stable orientation on timescales larger than the integration time. In contrast, a free diffusive rotation of the dye on the timescale of the integration time would lead to anti-correlation of the vertical and horizontal fluorescence signals. Anti-correlation (and therefore free rotation of the dye molecule) is likely caused by imperfections in the surface attachment of the stalled complexes. Finally, the fluorescence disappears in a single step at around time t = 22 s, indicating the presence of a single dye that was photobleached. These experiments (and an additional control discussed in Text S1), demonstrate that the polarization of the emitted fluorescence is an accurate reporter of the position of the connector protein, and that our instrument is indeed capable of detecting the changes in the fluorescence polarization, and hence the connector orientation, due to rotation on the single-fluorophore level. Constrained by the properties of single dyes and the camera, we can measure connector rotation rates from about 0.1–2.5 Hz, which corresponds to actual signal frequencies of 0.2–5 Hz due to the fact that the dipole emission has a 2-fold rotational symmetry. Current models for rotation predict a frequency within our detection bandwidth.

Simpson et al. [5] have proposed a model for connector rotation based on the symmetry of the capsid and the connector. The connector is a homododecamer that sits at the 5-fold portal vertex of the icosahedral capsid. This 12/5 symmetry mismatch dictates that the relative alignment between connector and capsid is recapitulated with every 12°-rotation between the two structures. Furthermore, in vitro packaging experiments have measured the DNA-packaging rate at saturating ATP to be ∼100 bp/s [2], and biochemical studies have shown that the step-size of the motor is 2 bp per ATP [8,43]. Given these figures, this rotation hypothesis predicts a rotation frequency of 1.65 Hz. Such rotation would result in a frequency of 3.3 Hz for the measured fluorescence signal in our experiment. Alternatively, if the connector were to track the helicity of the DNA [16], the rotation would be 36° per basepair and the resulting signal frequency would be ∼20 Hz. In our experiments, the packaging velocity was reduced 2- to 4-fold by simply reducing the ATP concentration [8] in order to reach a signal frequency below 5 Hz, which is within our experimental time resolution.

For actual packaging experiments, we illuminated the dye with homogeneously polarized light by time-sharing two different excitation polarizations. The emitted fluorescence was then detected in two orthogonal detection channels (see Materials and Methods for details). If the dye were to rotate in the plane parallel to the chamber surface around the DNA being packaged, the intensity of horizontally polarized light would oscillate according to a sine-squared function, while the vertically polarized intensity would oscillate in the same manner with a phase shift of 90°. The two channels, therefore, would show an anti-correlated modulation of the fluorescence intensity. (If the rotation axis is not perpendicular to the chamber surface, other phase shifts might be observed. We performed extensive simulations that suggested various limitations to our detection ability, and these are discussed below.)

**Figure 6:**
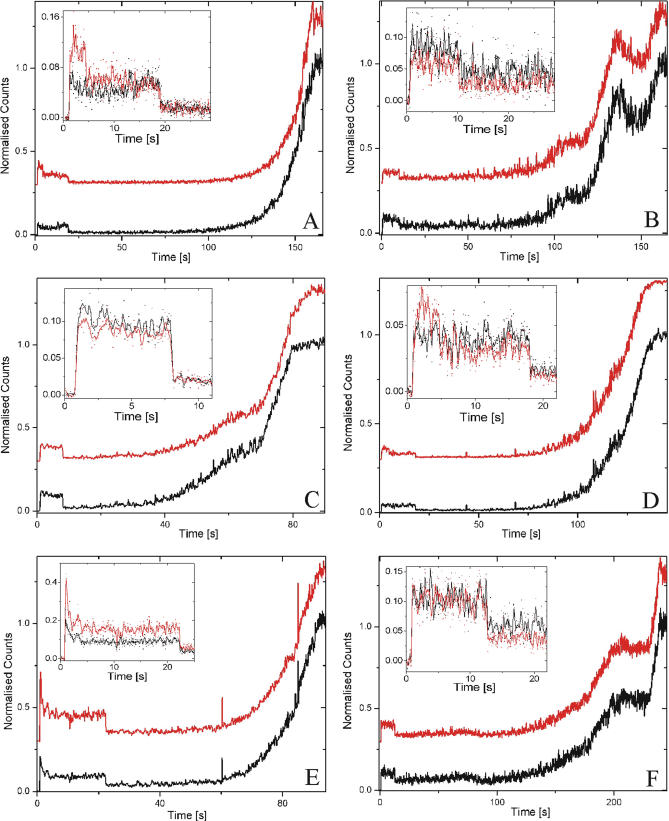
Fluorescence Signal from Packaging Complexes The graphs show the fluorescence intensity of vertical (black) and horizontal (red) polarization detected simultaneously. One example for each of the six investigated mutants is shown. The data were recorded with an integration time of 75 ms and three-points smoothing was applied. The camera background (closed shutter) was subtracted. The signal was normalized using the fluorescence intensity of the bead after packaging was completed. The traces are vertically shifted for clarity. The inset shows a zoom of the data. Here, the unfiltered data is displayed (scatter) together with the three-point sliding average. (A) Fluorescence recorded for a single complex of mutant 170C. The shutter is opened after about half a second. After t = 4 s the first dye bleaches and after 19 s the second. After about t = 100 s the fluorescence signal again starts to increase, since the magnetic bead is pulled into the evanescent field as the prohead reels in the DNA. The signal increases in an exponential fashion as the fluorescent bead samples the intensity profile of the evanescent wave. Similar behavior was observed for (B) 97C; (C) 168C; (D) 189C; (E) 190C; and (F) 260C.

Figure 6 shows typical examples of the single-molecule fluorescence signal during DNA packaging by the ϕ29 motor complex. Simultaneously, packaging activity is observed using a magnetic bead attached to the free end of the viral DNA. The red and black time traces show the fluorescence intensity detected in the two perpendicular polarization directions—horizontal and vertical, respectively. Figure 6A shows the signal for the mutant 170C. At t = 0.5 s, the signal of two single fluorophores can be seen. The first fluorophore bleaches after about 4 s, the second after about 19 s. This multi-step bleaching demonstrates our ability to quantify the number of dyes observed, and in rotation experiments only single-fluorophore traces were analyzed. The ratio of the intensities in the black (vertical) and red (horizontal) channel indicates that the first fluorophore is aligned almost horizontally, while the second fluorophore is at an angle of about 45° in between the two polarization directions. After about 100 s, scattered light from the magnetic bead, which is attached to the free end of the DNA, becomes visible in the trace. As the prohead continues packaging the DNA, the bead is pulled further into the evanescent field of the excitation by the green laser until it touches the surface at about t = 165 s. The packaging, assuming an approximately 10-kb tether, was therefore about 60 bp/s, consistent with bulk and optical tweezers measurements at this ATP concentration. It should be noted that we did not observe beads that were slowly pulled toward the packaging complexes in control experiments without ATP. Therefore, this behavior can be, without doubt, identified with the ATP-dependent DNA packaging of the motor complex. The center of the bead and the center of the initial single-dye fluorescence signal are within one pixel from each other, demonstrating the colocalization between the fluorophore and the packaging complex. By considering the density of fluorescent spots on the surface when using highly overlabeled proheads, we estimate that over 98% of such colocalized events are due to a tethered bead and dye molecule attached to the same packaging prohead (see Text S1 for details). We have recorded more than 50 of these single dye/bead colocalized traces from six different mutants (see Text S1 for a complete list).

The data were analyzed in various ways. Details are given in Text S1; in short: First we used a normalized sliding correlation function that measures the correlation of the two perpendicular signals over a window of variable size. If the motor rotated during packaging, our simulations (including noise) suggest that one out of four traces should give an average correlation coefficient less than −0.3 for many seconds, assuming a random dye orientation. We never observed such correlation coefficients for an extended period of time (several seconds) in any of the data collected (Text S1). Second, we checked for periodicities in the channels by looking at Fourier transforms and cross-correlations. Our simulations predict that motor rotation generates a periodicity in more than 90% of the traces in the accessible frequency range, assuming random orientation of the dye molecule relative to the axis of rotation and random orientation of this rotation axis with respect to the optical axis. We did not observe this periodicity in a single trace.

## Discussion

We have studied a possible rotation of the bacteriophage ϕ29 portal motor protein with respect to the capsid on the single-molecule level during DNA packaging. With six different connector mutants, we did not observe a single trace resembling a signal expected for connector rotation. These results permit us to rule out with very high probability (see below) the compressive-ratchet model for connector rotation proposed by Simpson et al. [5] that involves a rotation by 6° per basepair. At the experimental ATP concentration of 25–50 μM, this rotation would lead to a rotation frequency in the fluorescence signal of about 1–2 Hz, which is easily detectable with our instrument. Similarly, a rotation model in which the DNA is wrapped around the external surface of the connector, with rotation providing an indirect translocating force [1,44], would generate a rotation rate of 3° per basepair, also within the detection range of this experiment. A rotation rate below our detection limit is very unlikely, as a frequency of 0.1 Hz (our lower detection limit) would already correspond to as few as 0.6° per basepair—which would not fit any current model for rotation. On the other hand, we would be capable of observing a rotation of the connector if it were to follow the DNA double helix pitch in a nut and bolt fashion (10.5 bp per 360°), which would yield a rotation frequency in the fluorescence signal of 5 Hz at 25 μM ATP. While we can rule out complete rotation of the connector relative to the shell, we cannot rule out partial rotation over a small angle followed by return to the original position. In order to detect such transient rotation that would be consistent with models of connector flexure, polarization sensitive fluorescence correlation spectroscopy experiments would have to be performed.

There remains a small uncertainty about packaging motor rotation due to the unknown orientation of the dye molecule with respect to the putative rotation axis of the connector and due to the lack of absolute labeling specificity. Simulations of dye emission (unpublished data) show that there are orientations of the dye where neither a correlated nor an anti-correlated signal can be observed, even if the connector is rotating. Given the random orientation of the rotation axis with respect to the substrate and of the dye axis with respect to the rotation axis, this situation should happen in about one out of ten traces for the signal levels shown. Here, we reduced the likelihood of an unfavorable orientation of the dye molecule with respect to the putative rotation axis by investigating six different mutants. We cannot rule out that all mutants result in unfavorable dye molecule orientations, although we consider this possibility highly unlikely.

Considering the labeling specificity, there is currently no method to separate the connector protein (gp10) from the capsid protein (gp8) and re-assemble them again. Therefore, we have to label the connector in intact prohead particles, which might allow some dyes to attach to the nonrotating capsid. However, the fact that the capsid does not contain cysteines allowed us to achieve a specificity of more than 80% of dye on the connector of the 170C as observed on denaturing (SDS-PAGE) gels (Figure 3). We can also rule out that our selection of actively packaging complexes leads to a selection of nonlabeled complexes, since we have shown that labeling does not affect packaging efficiency or speed (Figure 3B and unpublished data). As a result, about four out of five of the observed dyes can be expected to be attached to the connector.

While no rotation of the connector was observed experimentally, does that mean that the connector does not rotate? In order to answer this question, we have performed a mathematical analysis of the remaining uncertainties. This analysis is described in great detail in Text S1. In brief, if one includes the uncertainty of unfavorable orientation, colocalization, labeling specificity, and rigidity of attachment, one can rule out connector rotation with more than 99% probability.

In our experimental design, we were able to eliminate several limitations of previous efforts to combine single-molecule fluorescence and magnetic tweezers [45]. Magnetic beads scatter a great deal of light and are therefore not compatible with most single-molecule fluorescence experiments. However, as demonstrated by our results, if one uses highly localized excitation fields, like an exponentially decaying fluorescence excitation used here, they can be combined with single-molecule fluorescence. For a processive motor like the bacteriophage ϕ29 packaging complex, the disadvantage of using spatially separated trapping and fluorescence detection can be overcome by the colocalization of single-molecule fluorescence and bead fluorescence after the bead has been pulled close to the surface. Our experiments have shown that beads kept at a distance of larger than ∼1 μm do not introduce a significant scattered signal at the Cy3 fluorescence wavelength. They do block part of the emitted fluorescence light, but for tethers longer than 1 μm and bead sizes of 1 μm, the detected dye fluorescence is calculated to decrease by less than 10%. Another advantage of this setup is the possibility of parallel observation. In some preparations, we could observe the packaging of more than five complexes simultaneously.

In previous single-molecule fluorescence studies on biological systems, the fluorescence signal itself was the only evidence of biologically relevant activity. Therefore, there had to be a characteristic and expected feature in the single-molecule fluorescence signal and sufficient statistics to confirm that the biological system is the cause for the observed signal. Obtaining these statistics can necessitate the observation of tens of thousands of fluorophores [33]. Here, we have presented a method that overcomes this problem. We can select for fluorophores that are attached to independently monitored active biological systems and observe their single-molecule fluorescence. In the present application, we can colocalize a fluorophore with a translocated bead. This leads to a 98% confidence that the observed fluorescence signal originates from a packaging prohead. The setup, therefore, opens exciting opportunities for the study of a number of different systems, such as RNA polymerase transcription initiation or elongation complexes, ribosomes, spliceosomes, or as shown here, nucleic acid translocation or packaging complexes in real time and with high resolution.

In conclusion, we were able to test the connector rotation hypothesis, a long-standing prediction of several DNA packaging models. Our single-molecule experiments exclude with very high probability (more than 99%) the predominant model that the connector rotates with respect to the capsid. Having established that the connector does not rotate during packaging, it is important to ask how DNA is driven into the capsid. A model consistent with all experimental data was proposed recently by Chemla et al. [8]. In this model, ATP binding, hydrolysis, and release of products induce conformational changes in the ATPases that are directly involved in the translocation of the DNA [8]. Specifically, the translocation step of the DNA is triggered by, or performed by, the ring of ATPases via a conformational change that follows release of phosphate after ATP hydrolysis [8]. Here, we add to this model the idea that the connector could function as a valve to prevent DNA from leaking out. The spring-like shape of the connector suggests, indeed, that through compression and expansion, the connector may act as a “Chinese finger trap” (George Oster, personal communication) allowing the passage of the DNA in one direction during packaging but preventing its exit in the reverse. A complete understanding of the coupling that occurs between the ATPase, the pRNA, and the connector substructures is needed to refine our picture of the molecular mechanism of this powerful motor. Finally, the possibility that translocation could be driven by a (nonrotating) compression/extension ratchet mechanism is an intriguing idea, but one for which there is no direct experimental evidence to date and that is distinct from the mode of action established for certain AAA+ -related ATPases.

## Materials and Methods

### Surface preparation.

For all experiments, we used quartz slides (Finkenbeiner Incorporated, http://www.finkenbeiner.com) cleaned in piranha at 60 °C overnight, rinsed with purified water (Barnstead, E-pure), sonicated in 2% Hellmanex (Hellma, http://www.hellma.com), rinsed again, sonicated, and stored in pure water. Slides were blown dry with nitrogen immediately before being placed in 1 ml vectabond (Vector Laboratories, http://www.vectorlabs.com) and 100 ml acetone for 5–10 min. The slides were then washed in 100 ml water, slowly pulled out of the water bath such that no water remained on the hydrophobic surface, and placed in a wet box.

100 μl of a mixture of 3 mg Biotin-PEG-NHS (Mw3400, Nektar Therapeutics, http://nektar.com), 80 mg mPEG-SPA (Mw5000, Nektar Therapeutics), and 550 μl 0.1 M bicarbonate buffer was then placed on each slide and kept in the dark for 3 h. Afterward, the slides were rinsed thoroughly with water, dried with nitrogen, and assembled into a flow chamber by placing a Nescofilm gasket in between the quartz slide and a cover slip and heating for 3 min at 100 °C.

The assembled chamber was then rinsed with 1 ml phosphate-buffered saline and incubated with 0.2 mg/ml streptavidin for about 20 min. After being rinsed again with phosphate-buffered saline and incubated with biotinylated antibodies against the capsid protein, gp8, (0.1 mg/ml) for 25 min, the chamber was rinsed with 1 ml 0.5× TMS (25 mM Tris-HCl, 50 mM NaCl, 5 mM MgCl_2_) and then with 250 μl of buffer XS (800 μl of 0.5× TMS, 10 μM ATP, 10 μM ATP-γS, 0.2 mg/ml BSA, 2 mg/ml glucose, 1% w/v beta-mercaptoethanol, 0.02 mg/ml catalase, 0.1 mg/ml glucose oxidase, and 0.8 μl of RNase inhibitor (SuperaseIn, Ambion, http://www.ambion.com).

### Protein preparation, mutation, and labeling.

With the exception of prohead particles, components for the ϕ29 in vitro packaging system (DNA-gp3, gp16, 120-base pRNA) were produced as previously described [46,47].

Prohead particles were produced in E. coli by overexpression of prohead structural proteins in HMS(DE3)pAR 7-8-8.5-9-10 [48]. Two wild-type cysteines (C76 and C265) in the ϕ29 connector, gp10, were replaced by serines using standard site-directed mutagenesis to produce a cysteine-free clone, and individual amino acids in gp10 were replaced with cysteines to produce a library of single-cysteine mutants. Particles were produced by induction of mid-log cultures grown in LB media with 0.5 mM IPTG for 2 h. Cells were pelleted and re-suspended in a lysis buffer containing 50 mM Tris HCl (pH 8.0), 20 mM NaCl, 2 mM EDTA, 2 mM DTT, and 10 mg/ml lysozyme. After a 20-min incubation to produce sphaeroplasts, MgCl_2_ was added to 4 mM final concentration and DNase was added to a final concentration of 10 μg/ml to digest cellular DNA. Complete lysis was achieved with the addition of deoxycholate to 0.25% w/v. Extracts were clarified by centrifugation, and prohead particles were purified on 10%–40% w/v sucrose zonal gradients (45 kilo-rotations per min, 45 min, 20 °C) in a SW55 rotor (Beckman) buffered with 1× TMS buffer (50 mM Tris-HCl, 100 mM NaCl, 10 mM MgCl_2_ [pH7.8]). Particles were collected, concentrated by pelleting, and re-suspended in 1× TMS.

Labeling was conducted by adding an equal volume of Cy3-maleimide in H_2_O to prohead samples to provide the appropriate molar amount of dye with respect to available connector cysteine. After labeling for 1 h at room temperature, particles were purified away from free dye by sucrose gradient sedimentation (5%–20% w/v sucrose in 1× TMS, 45 K, 30 min, 20 °C). Particles were pelleted and re-suspended as above. Particles were quantified and checked for purity and labeling efficiency by SDS-PAGE and for DNA-packaging activity (Figure 3).

### Stalled complex and bead preparation.

Labeled proheads were reconstituted with 120-base pRNA at a ratio of 10 pRNAs/prohead by mixing 2 μl of pRNA (0.07 mg/ml) with 2 μl of labeled proheads (1 mg/ml) for 15 min in 0.5× TMS. Reconstituted proheads were then added to a packaging reaction containing 2 μl of biotinylated DNA (0.44 mg/ml) and 2 μl of ATPase gp16 (0.025 mg/ml) in a final volume of 18 μl of buffered in 0.5× TMS (for a final ratio of two proheads:one DNA-gp3:15 gp16). After 5 min, packaging was initiated with 4 μl of ATP (250 μM). After 60 s, 2 μl of ATP-γS (1 mM) was added to stall the reaction. Magnetic beads (MyOne Dynabeads, Invitrogen, http://www.invitrogen.com) were prepared by washing three times in 0.5× TMS, then blocked by adding 2 μl of beads to 25 μl of 2 mg/ml BSA in 0.5× TMS. Blocked beads were then treated with 0.5 μl of RNAse inhibitor. Freshly prepared stalled complexes from above (4 μl) were bound to beads by mixing 0.1 μl of Superase Inhibitor, 3 μl of BSA (10 mg/ml), 0.5 μl of ApaLI (10 U/μl) in 80 μl of buffer X (0.5× TMS, 10 μM ATP, 10 μM γ-S ATP). After gentle mixing, the sample was incubated for 1 h for the ApaLI restriction digestion which cleaves at both the extreme right and left ends of the DNA, to reduce the presence of biotinylated DNA-gp3 on the bead surface not associated with stalled-packaging complexes, which can form nonspecific tethers due to the stickiness of gp3. (Left ends of packaged DNA-gp3 are in the prohead and protected from digestion.) Using a magnet, we washed the magnetic beads three times with buffer X. These washes removed free and cut DNA-gp3 ends, all free dye, and proheads that did not initiate packaging. Finally, the beads were flowed into the chamber and incubated for 10 min. To restart packaging, 0.5× TMS buffer containing 0.2 mg/ml BSA, 2 mg/ml glucose, 1% w/v beta-mercaptoethanol, 0.02 mg/ml catalase, 0.1 mg/ml glucose oxidase, and 50 μM ATP was flushed into the chamber.

### Instrument design.

During SMFP, the sample is illuminated in prism type total internal reflection geometry with a green laser (532 nm, CrystaLaser, http://www.crystalaser.com). There are two ways for a dye molecule to report on the rotation of a macromolecule: First, one could excite the dye molecule with linearly polarized light. The emitted fluorescence intensity would then be proportional to the square of the scalar product of dipole orientation and polarization direction. For a rotating dye molecule in the plane of the evanescent field, the emitted intensity would oscillate between a maximum and minimum within a given polarization as the molecule changes its orientation. A second option for using a dye molecule as a direction sensor is to illuminate with both horizontal and vertical polarization (with equal intensities, i.e., homogeneous polarization) but detect the polarization state of the emitted fluorescence. Here, the angle between emission dipole and polarizer in the detection path becomes important. For a molecule rotating in the polarization plane, the result would be an anti-correlated signal between the vertical and horizontal polarization. We chose to use the second option, since an anti-correlated signal cannot be confused with other events such as blinking or changes in molecular brightness. In order to achieve an illumination with homogeneous polarization, the light of the laser is coupled into an electro optical modulator (Linos Photonics, Incorporated, http://www.linos-photonics.com) that switches between two perpendicular polarization directions with a frequency of 10 kHz. This switch is orders of magnitude faster than the integration time during the experiment, and therefore, only homogenous polarization is observed. The two perpendicular polarizations are then split by a polarizing beam splitter (PBS). The resulting beams are both brought to the chamber in s-polarization, but from perpendicular directions—one from the side and one from the top. For this reason, we used a custom-made prism with two input ports. We checked the light intensity from both directions by comparing the signal scattered by beads attached to the flow chamber surface and adjusted the intensities to be equal in the center of the field of view; the intensities varied by less than 50% across the field of view.

The fluorescence light is collected by a high NA objective (Nikon, 1.2 NA, http://www.nikon.com) and separated in two perpendicular polarization components by a PBS. The two beams are spatially offset and recombined by another PBS. The two beams are then focused on a CCD camera (Cascade 512B, Photometrics, Roper Scientific Incorporated, http://www.roperscientific.com), such that two images, one for each polarization, can be read out simultaneously. One pixel on the camera (physical size 14 μm) corresponds to about 400 nm. Because of the huge dilution of phages, and therefore dyes on the substrate (less than one dye per 10 μm^2^), we used hardware binning by 3 × 3 pixels. Furthermore, since the fluorescent spot was not always centered on one point, we added up to four adjacent points for signal optimization. Two band-pass filters (580BP50, Omega Optical, http://www.omegafilters.com) are used to separate excitation and LED illumination from the single-molecule fluorescence signal. At the same time, the sample is illuminated with a red LED to observe the magnetic beads that are pulled away from the surface by two magnets. The red light scattered from the magnetic beads is separated from the fluorescence light with a dichroic mirror (630 DCLP, Omega Optical) and detected on a separate CCD camera (Watec, 902C; Watec Company, http://www.watec.com). In addition, an epi illumination can be used to focus onto the chamber surface without illuminating (and therefore bleaching) the surface.

## Supporting Information

Text S1 Supporting Online Text A rigorous assessment of the experimental uncertainties, and therefore, for the probability of nonrotation is described in the online text. The text includes, furthermore, a description of the statistical analysis, the results of this analysis, additional controls, and a table comparing the results of all six mutants.
(437 KB DOC)

## Abbreviations

ATP-γS: non-hydrolyzable ATP analog
gp: gene product
NA: numerical aperture
PBS: polarizing beam splitter
pRNA: prohead RNA
SMFP: single-molecule fluorescence polarization
