# Supplementary material for: Experimental Test of Connector Rotation during DNA Packaging into Bacteriophage ϕ29 Capsids
Source: PLoS Biol. 2007 Feb 20;5(3):e59. doi: 10.1371/journal.pbio.0050059 (PMC1800307; doi:10.1371/journal.pbio.0050059)
Supplement: Supplementary file 1 [file pbio.0050059.sd001.doc]

**Supporting Online Text**

The conclusions of the main paper are based on a rigorous assessment of the experimental uncertainties which is laid out in complete detail below.

Simulations of continuously rotating dye molecules (which will be published elsewhere) showed that for the experimentally determined signal to noise ratio a rotating molecule could be identified in more than 90% of all possible events. The remaining uncertainty arises from the fact that the dipole moment of the dye molecule could be oriented close to or perfectly parallel to the rotation axis, in which case rotation would not be detectable. However, assuming a random orientation more than 90% of the polarization time traces of the emitted fluorescence should show a periodically varying signal in one or both of the channels; in about 25% of the traces we should observe an anti-correlated signal (i.e. a phase shift of π).

Various sources of noise such as dye blinking, laser power fluctuations, and photon counting noise would generate signals that are uncorrelated or positively correlated. In contrast, the only feasible cause of a negatively correlated signal is the rotation or reorientation of the dye molecule. An anti-correlated signal over a certain period of time is a sufficient indicator of dye rotation. To check for anti-correlation we applied a standard sliding correlation function to our data. The correlation function is given in Equation S1.

$\text{Corr}1,2=\frac{1}{n\sigma1\sigma2}\sum_{i=1}^{n} (R1i-\mu1i)(R2i-\mu2i)$	(S1)

R_1i_ and R_2i_ are the measured values in the two polarization directions, respectively.

μ_1i_ and μ_2i_ are the mean values of the measured values and σ_1_ and σ_2_ are the standard deviations. This function was calculated for a sliding window of about 30 points which corresponds to 2.25 seconds. In addition, we applied a threshold criteria which was necessary in order to differentiate anti-correlation from random signal fluctuations. Anti-correlation was defined as an average correlation coefficient of less than -0.3 for a duration exceeding two seconds. The probability to detect a rotating dye-molecule with this function is already about 25%, for 18 traces the probability to detect at least one trace displaying anti-correlation would be larger than 99%.

Column A in Figure S1 shows the sliding correlation function of the six traces shown in Figure 6 of the main paper, namely examples of mutants 170C, 97C, 168C, 189C, 190C and 260C (from top to bottom). The sliding correlation function does not exceed the anti-correlation threshold in any of these traces. Furthermore, we did not find a single packaging complex that showed anti-correlation. Moreover, to check for any other phase shift, we calculated the cross-correlation function for the two perpendicular polarization directions (Equation S2) and its power spectrum.

$\text{XCorr}_{t}=\sum_{i=-n}^{n} R_{1i}R_{2i+t}$	(S2)

The results for the six traces from Figure 6 of the main paper are shown in column B and C of Figure S1, respectively. The number of points sampled during the lifetime of the dye is often too small for computing a meaningful power spectrum. Therefore, we catenated four repeats of the measured signal of each dye molecule in order to calculate the power spectrum. This procedure yields a good sampling of the frequency range of interest, but in turn produces peaks at very low frequency (around 0.05 Hz). Those peaks in trace one, four and five from the top correspond to a period of about 20 seconds, which is longer than the lifetime of the dye and therefore an artifact of the catenation. The peak from the mutant 168C (third trace from top) has a period of 8 seconds, which corresponds very well to the lifetime of the dye and is therefore also no indication of rotation.

While no signal of rotation was observed for any packaging complexes, the statistical analysis of the control experiments (compare to Figure 5 and description in main paper) demonstrate the viability of the method (Figure S2). Here, again we show the sliding correlation function, the cross-correlation as well as the power spectrum of the cross-correlation in A, B and C, respectively. The excitation polarization was rotated with a frequency of about 0.7 Hz; this frequency is found very clearly in the power-spectrum of the cross-correlation function (please note the almost one order of magnitude larger scale of the power-spectrum, as compared to the scale in figure S1C).

The rigidity of the dye-molecule attachment is crucial to the experiment, since the dye-molecule is used as a probe for the connector orientation. Additional evidence for this rigidity is presented in Figure S3. Here, the fluorescence signal of a phage labeled with two dye molecules is shown. We used conditions for the connector labeling resulting in rather low labeling efficiencies of on average less than one dye per prohead. However, we did observe a handful of events with a two-step bleaching, indicating the presence of two dye molecules.

The two dye molecules have different contributions to the two perpendicular polarizations, indicating that the orientation of the molecules is both, distinct and fixed on the timescale of the experiments (see Figure caption for details).

Estimate for the probability of non-rotation:

The probability for non-rotation is estimated from the following four arguments:

1.) Co-localization is defined as an observed dye (the single molecule signal at the beginning of a fluorescence trace) being attached to the same phage that packages the DNA attached to the observed bead (the exponentially rising signal at the end of a fluorescence trace). Due to the fact that not all complexes are labeled, as well as that not all complexes are packaging, there is a possibility that such co-localization arises not from a single complex, but from a pair of complexes. One of the complexes would have to be labeled, but not packaging, and the other would have to be not-labeled, but packaging. The probability for this co-localization is estimated in the following way:

We assume that the distance between phage proheads on the surface follows a Gaussian-Distribution. The average distance between proheads is estimated by using heavily labeled proheads (about 10 dye molecules per prohead) following the same experimental procedures as for the experiments with under-labeled particles. These experiments yielded an average distance of proheads of 5 points (a point is 3x3 pixels – see Instrument Design in main text for details) and a standard deviation of 1.5 points (square root of half the average distance). As a bead will not be co-localized if its final distance to the dye is more than 2 points, we obtain a confidence of 98% with the Normalized Gauss Distribution given in Equation S3 (x_1_=0, x_2_=2, μ=5, σ=1.5).

$p(t)=\frac{1}{\sigma\sqrt{2\pi}} \int_{t1}^{t2} e^{-t^{2}2}\sigma\text{dt}$	(S3)

With t_i_=(x_i_-μ)/σ and the standard deviation σ and the mean μ.

2.) Given the experimentally determined signal to noise levels and assuming a random orientation of the dye molecule with respect to the rotation axis, 90% of the molecules should show a periodically varying signal in one or both polarizations. This result was obtained in extensive simulations, which will be published elsewhere.

3.) The labeling specificity of the dye to the connector is not 100%. We estimated the labeling specificity using SDS Page electrophoresis and a high resolution gel imager (see main text for details). The labeling specificity varied between the mutants investigated (see Table S4 for details). For the 170C mutant the specificity was 80% (see Figure 3A in main text).

4.) Another important factor in estimating the overall fidelity of the experiment is the rigidity of the attachment. For this estimate, we attached stalled packaging complexes to the surface of the flow-chamber and rotated the excitation polarization. In 50% of the traces we find a correlated signal between the two detected perpendicular polarization directions, which is expected for a fixed dye molecule. In about 50% of the traces we see an anti-correlated or uncorrelated signal which can be explained by a dye molecule that is free to orientationally diffuse or rotate faster than the time scale of the integration time, but hindered on the time scale of the fluorescence lifetime. Most likely this sub-population is caused by imperfections in the surface attachment, such as only single antibody attachments, yielding complexes that are free to re-orient during the time scale of the experiment. We conclude from this control that the probability that a dye molecule has a fixed orientation when the phage is not packaging is 50%.

In order to find an estimate of overall fidelity, we assume that each aforementioned probability is independent. Multiplying the four probabilities outlined above gives a fidelity for non-rotation of Q(non-rot_event_) ~ 35 % for the mutant 170C. We did not observe such a signal in any of the experiments. In other words, the probability that the connector rotates despite the absence of a periodically varying signal for any trace is P(rot_event_)= 1- Q(non-rot_event_) ~ 65 %. Therefore, in order to estimate the probability that the connector might rotate in spite of our experimental data one simply needs to compute the overall probability:

P(rot_total_) = [P(rot_event_)] ^number of events^

For the case of the 170C mutant we observed 18 co-localized packaging events and therefore P(rot_total_)_170C_ < 0.001. Therefore we can exclude rotation with a probability Q(non-rot_total_) = 1-P(rot_total_) > 99%.

As mentioned in the main text, we cannot exclude that the dye on the mutant 170C is oriented in an unfavorable direction. Therefore, we investigated five more mutants at various positions and orientations in the connector (see Figure 2 of main text). An overview of the data and probabilities for each single mutant investigated is given in Table S4. None of the traces showed any hint towards rotation. The probability for the five mutants other than 170C is only lower because of the smaller sample size. We thus conclude that the connector of the φ29 portal packaging motor does not rotate during packaging.

**Supporting Online Figure Captions**

**Figure S1:** **Statistical analysis of the single-molecule data.**

Normalized sliding correlation function with n=30 (A), cross-correlation (B) and power spectrum (C) for the traces from Figure 6 in the main paper. The traces are from top to bottom of mutants 170C, 97C, 168C, 189C, 190C and 260C.

**Figure S2: Statistical analysis of the control.**

Normalized sliding correlation function with n=30 (A), cross-correlation (B) and power spectrum (C) for the control data from Figure 5 in the main paper.

**Figure S3: Fluorescence signal of packaging complex labeled with two dye molecules.**

A) Fluorescence signal of a packaging phage (260C) that was labeled with two dyes. The two dyes bleach at different times – the first at about 7s and the second at about 11s. The dye that bleached first can be seen to have contributed mostly to the intensity in the vertical polarization (black), whereas the longer-lived dye yields comparable intensities in both channels. Such an example shows that we can discriminate between different dye orientations.

B) The sliding correlation function shows no sign of rotation.

**Table S4:**

Number of co-localized single molecule traces (# of traces), estimated labeling specificity (label specificity), probability for one event showing rotation if the connector would rotate (Q(non-rot_event_)) and total probability for non-rotation (Q_total_) for all the different investigated mutants. Note: The probability for the five mutants other than 170C is only considerably lower because of the smaller sample size.

**Supporting Online Figures**

**Figure S1:**

**Figure S2:**

**Figure S3**

**Table S4**

| Mutant | # of traces | label specificity | Q(non-rot_event_) | Q_total_ |
| --- | --- | --- | --- | --- |
| 170C | 18 | 80 | 0.33 | > 99% |
| 189C | 9 | 80 | 0.31 | 98% |
| 190C | 9 | 80 | 0.26 | 98% |
| 168C | 8 | 70 | 0.31 | 95% |
| 260C | 7 | 70 | 0.31 | 93% |
| 97C | 5 | 60 | 0.26 | 80% |
